# Photography-based method for assessing fluorescein clearance test in dogs

**DOI:** 10.1186/s12917-018-1593-y

**Published:** 2018-09-03

**Authors:** Arianne Pontes Oriá, Miriam Flores Rebouças, Emanoel Martins Filho, Francisco de Assis Dórea Neto, Ana Cláudia Raposo, Lionel Sebbag

**Affiliations:** 1 School of Veterinary Medicine and Zootechny, Federal University of Bahia, UFBA, Salvador, Bahia Brazil; 2 Department of Veterinary Clinical Sciences, Iowa State University, College of Veterinary Medicine, Ames, Iowa USA

**Keywords:** Dogs, Fluorescein clearance test, Schirmer tear test, Tear film

## Abstract

**Background:**

The fluorescein clearance test (FCT) provides insight into the tear film dynamics. The purpose of this study was to describe an inexpensive and practical method for assessing FCT in dogs, using photography and software analysis, and to assess the retention time of 1 vs. 2 eye drops on the canine ocular surface.

**Methods:**

(i) In vivo - Eight healthy German Shepherd dogs were recruited. Following topical anesthesia with 0.5% proxymetacaine, each eye sequentially received (1 week apart) either 1 drop (35 μL) or 2 drops (70 μL) of 0.5% fluorescein. A Schirmer strip was inserted in the ventral conjunctival fornix for 10 s at the following times: each 10 min for 100 min, 24 h, 48 h and 72 h. (ii) In vitro - Schirmer strips were placed for 10 s in contact with microplate wells containing 1 or 2 drops of 0.5% fluorescein. In both experiments, the fluorescein-impregnated Schirmer strips were immediately imaged, and the area and intensity of fluorescein uptake were analyzed with ImageJ software. For the in vitro experiment, images were evaluated by the same examiner (repeatability) or two examiners (reproducibility).

**Results:**

Photography-based FCT was easy to perform and showed high repeatability and reproducibility (coefficients of variation ≤2.75%). In vivo, the area and intensity of fluorescein uptake on Schirmer strips were significantly greater at 30 min and 40 min post- fluorescein instillation in the 2 drops vs. 1 drop groups (*p* ≤ 0.044). Compared to baseline, the residual fluorescein uptake on Schirmer strips was < 5% at 60 min and 90 min in the 1 drop and 2 drops groups, respectively.

**Conclusions:**

Photography-based FCT is a practical and reliable diagnostic tool with various clinical and research applications in veterinary medicine. Instillation of two drops provided greater amount and longer retention on the anesthetized canine ocular surface than a single drop. Fluorescein clearance time of a single drop in dolichocephalic dogs is 60 min.

## Background

The ocular surface is covered by a thin layer of tear film, mostly responsible for lubrication, nutrition, and protection against microbial and toxic agents [1]. Tears are secreted by the lacrimal glands, spread onto the corneo-conjunctival surface, then either evaporate, absorb into tissues of the ocular surface, or drain through the nasolacrimal duct. Together, these physiological aspects (secretion, distribution, turnover and elimination) are termed ‘tear film dynamics’ and are critical for the health and function of the eye [1]. Tear film dynamics can be assessed by various modalities including interferometry, lacrimal scintigraphy and fluorescein clearance test (FCT) [1].

FCT relies on measuring the decay of fluorescein in the tear film over a period of time. It is often performed with a commercial fluorophotometer device that can quantify fluorescein concentrations in a sensitive manner [2]. However, the fluorophotometry technique requires costly equipment and is not practical for routine clinic use. Thus, a simpler clinical test has been developed [3]. It involves photography of Schirmer strips to monitor dye disappearance, and has been reported to have excellent correlation with the fluorometric assessment of tear clearance in humans [3].

The main purpose of the study was to describe the Schirmer-based FCT in healthy dogs. Further, we aimed to determine the retention time of one vs. two drops on the canine ocular surface, thus describing a practical application of the FCT in veterinary species.

## Methods

### In vivo evaluation

#### Animals

Eight healthy intact German shepherd dogs (6 males and 2 females), aged 3 to 5 years (3.9 ± 0.83 years) were recruited from the Military Police of Bahia, Brazil. All dogs were confirmed to be ophthalmoscopically healthy with patent nasolacrimal ducts by slit lamp examination, indirect funduscopy, rebound tonometry, Schirmer tear test, fluorescein staining of the ocular surface, and Jones test (fluorescein passage test).

#### Experimental design

A test solution of 0.5% fluorescein was made by 1:1 dilution between 1% fluorescein solution^1^ and *0.5%* c*arboxymethylcellulose sodium* lubricating solution.^2^ Based on findings of a preliminary study using an automatic pipette^3^ to measure the drop size of 5 ophthalmic solutions commonly used in Brazil, the volume of a drop was set to 35 μL for the experiments described below (data were not shown).

**Fig. 1:**
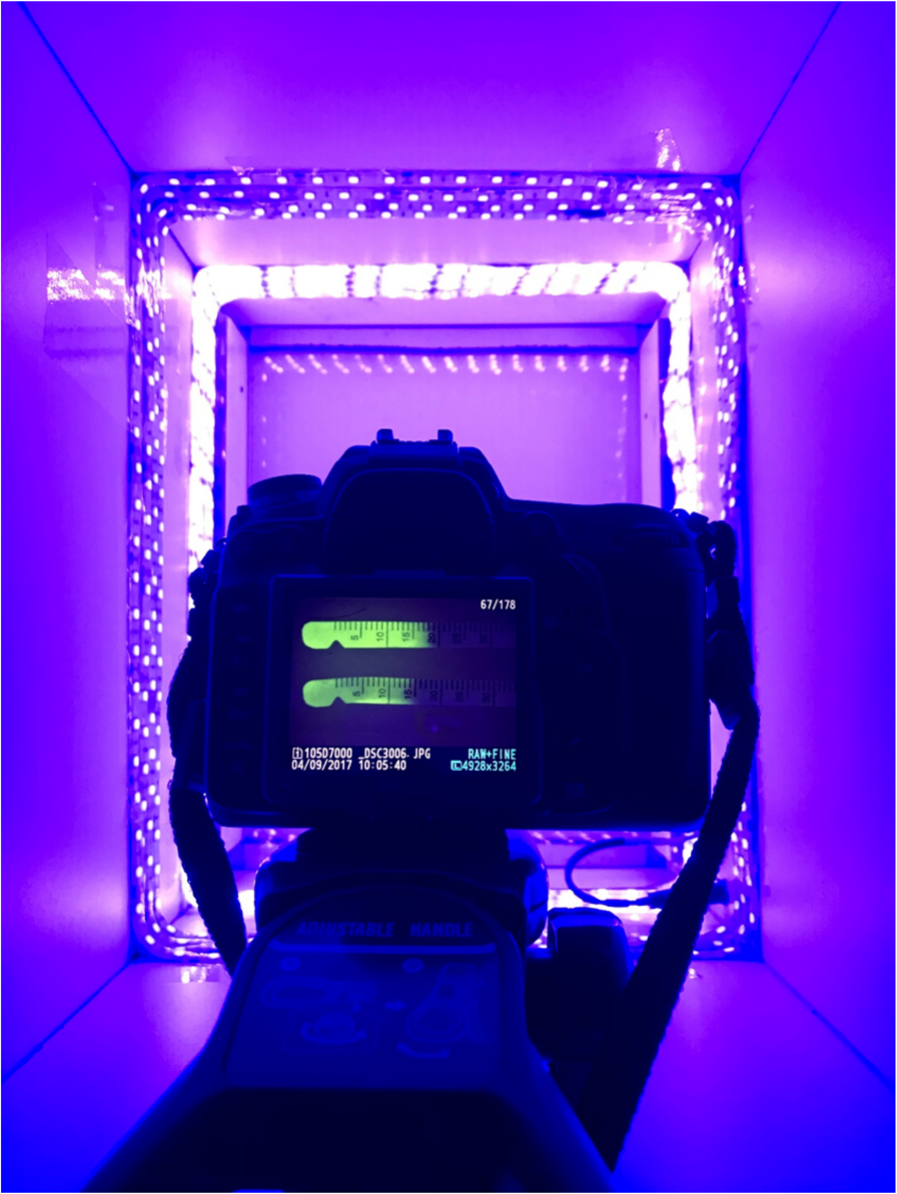
Equipment set up to capture the fluorescence in Schirmer tear strips immediately after collection from the eyes. A camera with yellow filter is placed 23 cm above Schirmer strips in a box illuminated with blue light

The study was conducted in the morning hours, with a temperature of 27.9–38.0 °C and an ambient humidity of 38–55%. Schirmer tear test I (STT-I) was performed in each eye by inserting a standard Schirmer strip^4^ in the lower lateral conjunctival fornix for 1 min. Ten minutes later, a drop of 0.5% proxymetacaine hydrochlorid^5^ was instilled in each eye, and after removing the excess solution with a sterile swab, Schirmer tear test II (STT-II) was performed and recorded in mm/min. Ten minutes later, an automatic pipette was used to instill 35 μL (1 drop) of 0.5% fluorescein in the left eye and 70 μL (2 drops) in the right eye. The excess of fluorescein solution was gently removed from the conjunctival sac with a sterile swab. At *t* = 10 min and every 10 min thereafter for 100 min, as well as 24 h, 48 h and 72 h, a Schirmer strip was inserted into the lateral lower conjunctival fornix of each eye for 10 s. Immediately after removal from the conjunctival fornix, the Schirmer strips were placed in a box and a Nikon camera^6^ with a yellow filter^7^ placed 23 cm above the strips was used to picture the tear fluorescence in a blue light^8^ (Fig. 1). The protocol was repeated in each animal after 1 week of washout period, instilling 35 μL (1 drop) of 0.5% fluorescein in the right eye and 70 μL (2 drops) in the left eye (crossover study design).

### In vitro evaluation

The 0.5% fluorescein solution was transferred to a 96-well microplate,^9^ 15 wells containing 35 μL (1 drop) and 15 wells containing 70 μL (2 drops). Schirmer strips were placed in contact with the wells for 10 s, then immediately transferred to the box and photographed as described in the in vivo experiment.

### Data analysis

**Fig. 2:**
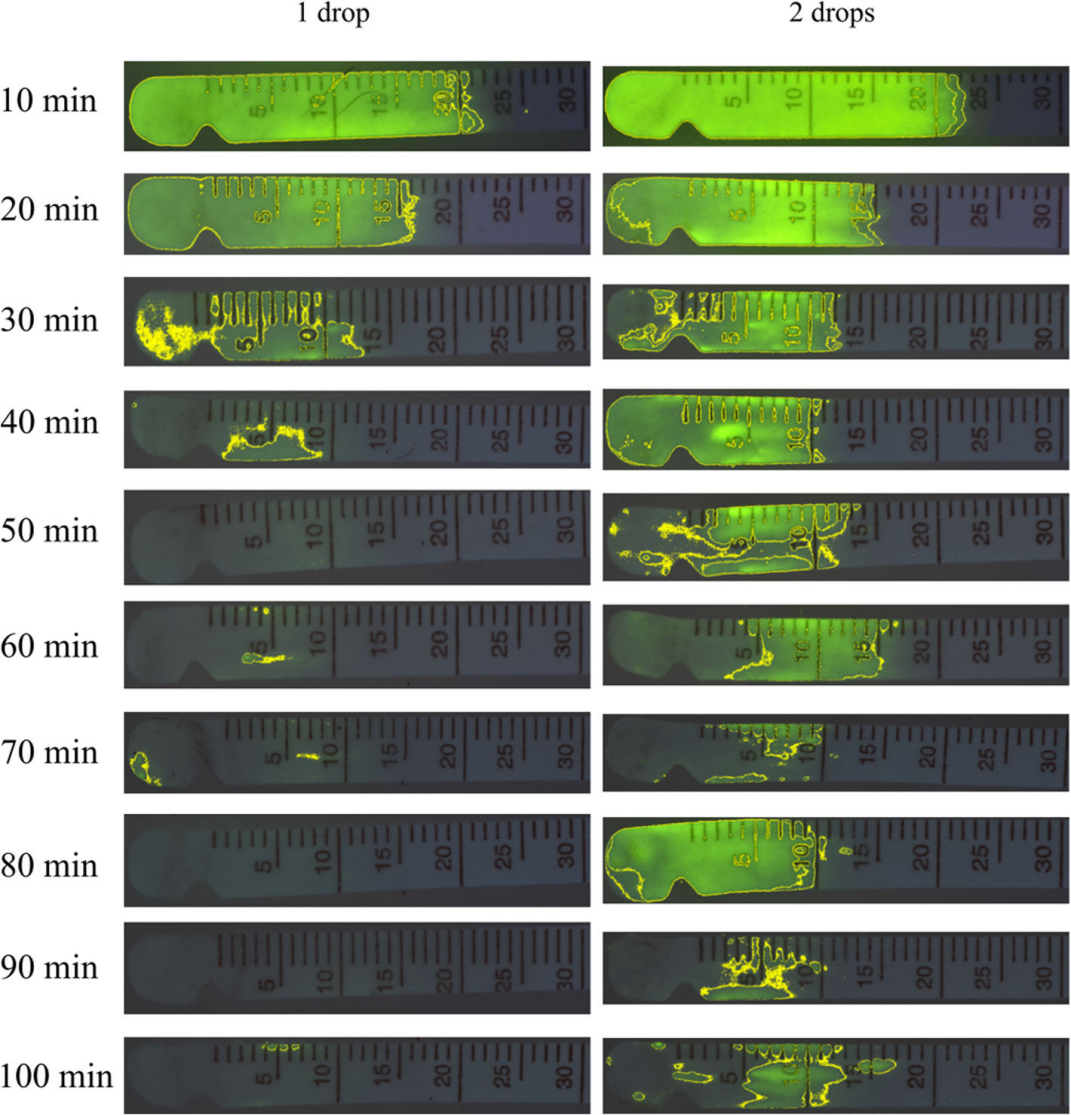
Representative analysis by ImageJ software of a Schirmer strip impregnated with fluorescent tear fluid. In this example, the area of fluorescein was 155 mm^2^ and the intensity of fluorescence was 1828 AU

All pictures were analyzed with the ImageJ Program^10^ [4], recording the following two measure outcomes in each Schirmer strip (Fig. 2): area of fluorescein uptake (mm^2^) and fluorescein intensity (arbitrary units; AU). The areas of fluorescein uptake were automatically delineated by the software although slight manual adjustments were needed on selected cases.

For the in vitro study, the same image was analyzed twice by the same examiner (intra-examiner variability, i.e. repeatability) or by two different examiners (inter-examiner variability, i.e. reproducibility), evaluating the area of fluorescein uptake and fluorescein intensity as described above. The coefficient of variation (CV, expressed in %) was calculated for assessing test repeatability and reproducibility.

Normality of the data was assessed with the Shapiro-Wilk test. The Wilcoxon signed-ranked test was used to compare STT-I and STT-II values in the same eye and to compare the right and left eyes for STT values, area of fluorescein uptake and fluorescein intensity. Differences in fluorescein areas and fluorescein intensities between one drop and two drops groups were examined with the Friedman test followed by Dunn’s pairwise comparisons. The same test was used to compare the residual fluorescein areas and intensities between both groups, calculated at each time as a percentage compared to the initial data collected (i.e. 10 min post fluorescein instillation). Statistical analysis was performed using GraphPad Prism,^11^ and values *p* < 0.05 were considered statistically significant.

## Results

### In vivo evaluation

**Fig. 3:**
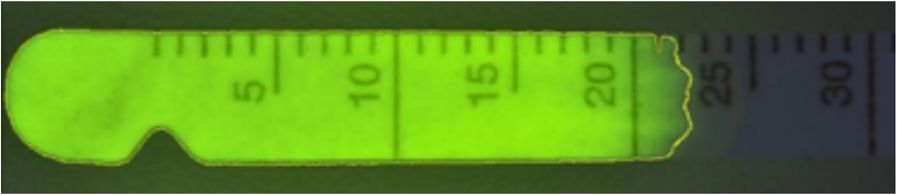
Sequential Schirmer strips collected from one German Shepherd dog at 10 min intervals from 10 min to 100 min following topical administration of either 1 drop or 2 drops of 0.5% fluorescein solution

Data was not normally distributed for any test (*P* < 0.05), so all results are presented as median ± semi-interquartile range. Results of STT-I testing (30 ± 3.6 mm/min) were significantly greater (*p* = 0.004) than STT-II values (28.2 ± 3.8 mm/min). The FCT was well tolerated in all dogs. An example of fluorescein strip analyzed by ImageJ is depicted in Fig. 3, in which the fluorescein area was 155 mm^2^ and the fluorescein intensity was 1828 AU.

**Fig. 4:**
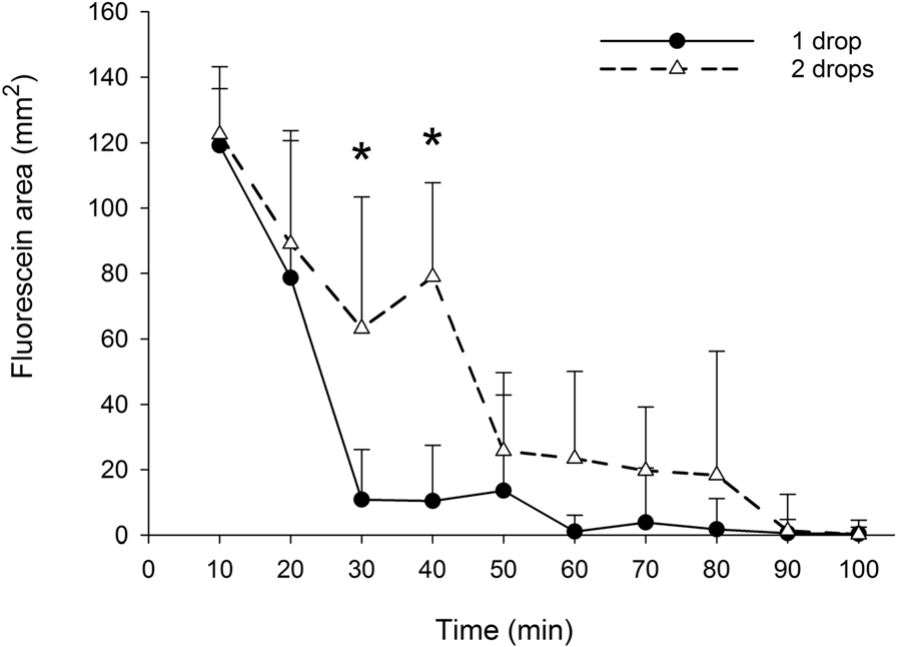
Median ± semi interquartile range of fluorescein area obtained from 8 German Shepherd dogs after topical administration of either 1 drop or 2 drops of 0.5% fluorescein solution. An asterisk (*) represents statistically significant difference between the 1 drop and 2 drops groups

**Fig. 5:**
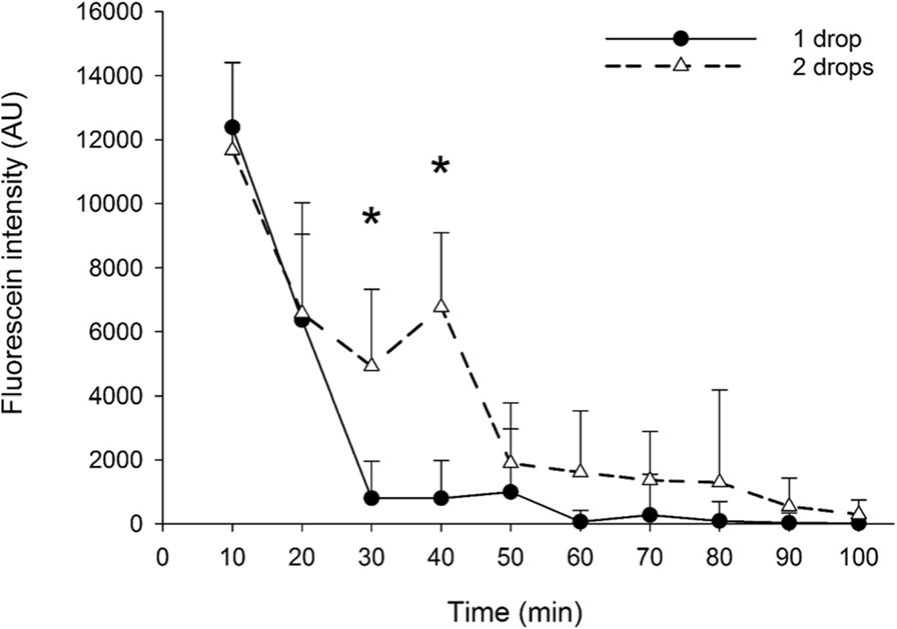
Median ± semi interquartile range of fluorescein intensity obtained from 8 German Shepherd dogs after topical administration of either 1 drop or 2 drops of 0.5% fluorescein solution. An asterisk (*) represents statistically significant difference between the 1 drop and 2 drops group

The area and intensity of 0.5% fluorescein impregnated onto the Schirmer strips decreased over time (Figs. 4 and 5). At 30 min, eyes that were administered two drops vs. one drop of 0.5% fluorescein solution yielded Schirmer strips that had significantly greater fluorescein areas (63.1 ± 40.3 and 10.8 ± 15.4 mm^2^, respectively; *P* = 0.025) and fluorescein intensities (4916.6 ± 2418.6 and 796.0 ± 1163.1 AU, respectively; *P* = 0.044). The same finding was noted at 40 min for both fluorescein areas (79.0 ± 28.8 and 10.4 ± 17.1 mm^2^, respectively; *P* = 0.039) and fluorescein intensities (6766.9 ± 2324.0 and 798.7 ± 1183.2 AU, respectively; *P* = 0.002).

Although the fluorescein areas and intensities were overall greater in the 2 drops group compared to the 1 drop group during other timepoints, differences were not statistically significant (*p* ≥ 0.078). Table 1 describes the residual fluorescein areas and intensities at each time point (times 20–100 min) for each group, calculated as a percent relative area or intensity compared to the first data collected (time = 10 min). Despite noticeable differences in the median values between both groups, no statistical significances were noted at any time point for either fluorescein area (*P* = 1.000) or fluorescein intensity (*p* > 0.441).

**Table 1:** Median ± semi interquartile range residual area and intensity uptake over time in healthy German shepherd dogs that received 1 drop or 2 drops of 0.5% fluorescein solution

	*Fluorescein area (%)*		*Fluorescein intensity (%)*	
Time	1 drop	2 drops	1 drop	2 drops
*20 min*	61.5 ± 35.1	70.7 ± 28.7	53.5 ± 25.7	52.6 ± 17.6
*30 min*	12.4 ± 17.3	50.8 ± 26.4	8.7 ± 10.8	40.6 ± 21.4
*40 min*	9.1 ± 17.8	60.7 ± 32.1	6.0 ± 11.4	49.9 ± 19.5
*50 min*	18.6 ± 28.0	20.4 ± 22.0	12.5 ± 16.3	13.4 ± 14.7
*60 min*	0.8 ± 6.2	24.4 ± 21.3	0.4 ± 3.5	13.5 ± 14.0
*70 min*	4.1 ± 14.4	17.2 ± 15.4	2.5 ± 8.1	9.7 ± 12.7
*80 min*	1.3 ± 9.4	16.5 ± 29.5	0.7 ± 5.3	12.4 ± 20.0
*90 min*	0.4 ± 3.6	1.3 ± 10.5	0.3 ± 2.0	4.6 ± 7.2
*100 min*	0.2 ± 1.8	0.2 ± 3.2	0.2 ± 1.1	2.5 ± 4.2

Residual fluorescein is calculated as a percentage compared to the initial data collected (i.e. 10 min post fluorescein instillation)

Twenty-four hours after dye instillation, fluorescein was still noticeable on Schirmer strips in 1/16 eyes (6.25%) of the 1 drop group and 6/16 eyes (37.5%) of the 2 drops group. At 48 h, fluorescein was present in 1/16 eyes (6.25%) and 2/16 eyes (12.5%) of the 1 drop and 2 drops groups, respectively. At 72 h, none of the Schirmer strips had fluorescein uptake, indicating that 3-day washout period is a minimum for FCT in dogs.

### In vitro evaluation

The coefficients of variation of intra-examiner and inter-examiner assessments were very low (≤ 2.75%), as summarized in Table 2, indicating excellent test repeatability and reproducibility.

**Table 2:** Median ± interquartile range of the coefficients of variation (CV) in measuring the area and intensity of fluorescein uptake in ImageJ by the same examiner (intra-examiner variability, i.e. repeatability) or two different examiners (inter-examiner variability, i.e. reproducibility)

	1 drop		2 drops	
Coefficient of variation (%)	Area of fluorescein uptake	Intensity of fluorescein uptake	Area of fluorescein uptake	Intensity of fluorescein uptake
Intra-examiner (repeatability)	2.01 ± 3.42	2.01 ± 3.03	1.20 ± 1.66	1.20 ± 1.94
Inter-examiner (reproducibility)	2.75 ± 2.78	2.38 ± 3.80	1.55 ± 2.70	1.59 ± 2.52

## Discussion

The present study describes a practical and cost-effective method to evaluate FCT in dogs. This test provides insight into the tear film dynamics and can be a valuable addition in diagnosis and management of ocular surface disease in veterinary species. In human patients, FCT has been shown to have a greater predictive value for ocular irritation than Schirmer tear testing and it correlates better with decreased corneo-conjunctival sensation and meibomian gland dysfunction [5, 6].

The technique used herein is a modification of previous reports in humans [3, 6, 7]. First, the duration of Schirmer strip insertion at each time point was set to 10 s, in contrast to 1 min [7, 8] or 5 min [3] in human subjects. Since standard Schirmer tear testing is performed over 5 min vs. 1 min in humans and dogs, respectively [9, 10] the duration of FCT was adjusted accordingly to avoid depleting the majority of the fluorescein dye from the canine ocular surface at the first time points. Second, our method employed standardized imaging technique in which a camera with yellow filter was set to a fixed distance from the Schirmer strips and a blue light was used to excite the fluorescein dye. The images were then analyzed objectively using the ImageJ software, thus representing an advantage over previous reports in which the fading of fluorescein dye was assessed subjectively with the naked eye [7], comparing the intensity of staining to a standard Schirmer color plate [3].

The main advantage of the present FCT over fluorophotometric evaluation [3] is the use of equipment readily available to most investigators. However, it may not be as sensitive as fluorophotometry to detect low concentrations of fluorescein, and one should only use the same type of Schirmer strips [11] of the same lot number [12] to reduce the variability in strip absorption of fluid.

The amount of fluorescein retained on the canine ocular surface was greater when 2 drops (70 μL) rather than 1 drop (35 μL) were instilled at baseline, with a statistically greater fluorescein area and intensity noted at 30 min and 40 min. The human palpebral fissure is capable of holding only 25–30 μL of fluid [13] so any excess would rapidly escape through the nasolacrimal duct or spill over the lower eyelid. However, this information is lacking in dogs, and it is possible that the palpebral fissure of a large German Shepherd dog can accommodate larger volumes of fluid. In beagle dogs, Gelatt and colleagues [14] noted that only 0.139% of fluorescein was retained on the ocular surface after 45 min of dye instillation, an amount much lower than the 8.7% noted in the present study. Differences in methodology notwithstanding, this variation between species warrant further investigation and may be partly explained by differences in nasolacrimal duct transit among brachycephalic, mesaticephalic and dolichocephalic dogs [15].

The homogenous population selected (all German Shepherd breed) represent a limitation of the study, as well as the relatively low number of subjects examined, although the cross-over study design improved the experiment’s statistical power. The use of topical anesthetic in FCT may also represent a limitation of the protocol, as it has been shown to suppress the rate of tear turnover in rabbits and thereby increase the pre-corneal residence time of ophthalmic solutions [16]. Thus, the differences noted between one vs. two drops should be interpreted with caution as these findings may differ in the non-anesthetized eye. Similar to human reports, topical anesthetic was used herein to reduce reflex tearing and thereby improve the reliability of Schirmer testing, and because tear clearance rates in anesthetized eyes are supposedly the same as basal tear turnover in non-anesthetized eyes [3]. However, the impact of topical anesthetic on Schirmer testing is not straightforward, with some authors reporting a greater variability of Schirmer values in anesthetized vs. non-anesthetized eyes [17].

## Conclusions

FCT represents a diagnostic tool with clinical and research potential in veterinary medicine. Clinically, FCT could be used to assess the clearance of tear film in patients with suspected aqueous tear deficiency or nasolacrimal duct obstruction. Based on our findings, clearance would be defined as normal (in dolichocephalic dogs) if a single drop of 0.5% fluorescein could not be detected (< 5% area and intensity) at the 60 min time point. Of note, this duration is longer than the 20 min timeline reported in human subjects [18]. In a research setting, FCT could have multiple applications such as determination of tear turnover rate in various breeds and investigation of the retention time of drugs that have different properties (e.g. viscosity, pH, etc.).

## Abbreviations

ARVO: Association for Research in Vision and Ophthalmology
AU: Arbitrary units
FCT: Fluorescein clearance test
STT-I: Schirmer tear test I
STT-II: Schirmer tear test II

## Footnotes

1 Drogavet®, Salvador, BA, Brazil

2 Ecofilm®, Latinofarma, São Paulo, SP, Brazil

3 Automatic pipette, Kasvi, Curitiba, PR, Brazil

4 Ophthalmos®, São Paulo, SP, Brazil

5 Anestalcon®, Alcon, São Paulo, SP, Brazil

6 D7000, ISO 200, f40, 2 s

7 Yellow filter, Hoya HMC Y [K2], California, USA

8 Led light, 12v, wavelength 470 nm, LedSafe®, São Paulo, SP, Brazil

9 96-Well Plates, ThermoFisher Scientific, Waltham, MA, USA

10 Image J, Rasband, W.S., ImageJ, U. S. National Institutes of Health, Bethesda, MD, USA, https://imagej.nih.gov/ij/, 1997–2016

11 GraphPad Prism version 7.00 for Windows, GraphPad Software, La Jolla, CA, USA, www.graphpad.com

## References

[1] Tomlinson A, Khanal S. Assessment of tear film dynamics: quantification approach. Ocul Surf. 2005; 10.1016/S1542-0124(12)70157-X. 10.1016/s1542-0124(12)70157-x 17131012

[2] van Best JA, Benitez del Castillo JM, Coulangeon LM. Measurement of basal tear turnover using a standardized protocol. European concerted action on ocular fluorometry. Graefes Arch Clin Exp Ophthalmol. 1995; 10.1007/BF00177778. 10.1007/BF00177778 7721117

[3] Xu KP, Tsubota K. Correlation of tear clearance rate and fluorophotometric assessment of tear turnover. Br J Ophthalmol. 1995; 10.1136/bjo.79.11.1042. 10.1136/bjo.79.11.1042 PMC505325 8534651

[4] Labno C. Basic Intensity Quantification with ImageJ. In. University of Chicago. http://rsbweb.nih.gov/ij/docs/examples/index.html -- Accessed 18 July 2018.

[5] Afonso A. Correlation of tear fluorescein clearance and schirmer test scores with ocular irritation symptoms, historical image. Ophthalmology. 1999; 10.1016/S0161-6420(99)90170-7. 10.1016/S0161-6420(99)90170-7 10201606

[6] Macri A, Rolando M, Pflugfelder S. A standardized visual scale for evaluation of tear fluorescein clearance. Ophthalmology. 2000; 10.1016/S0161-6420(00)00101-9. 10.1016/s0161-6420(00)00101-9 10889108

[7] Prabhasawat P, Tseng SCG. Frequent association of delayed tear clearance in ocular irritation. Br J Ophthalmol. 1998; 10.1136/bjo.82.6.666. 10.1136/bjo.82.6.666 PMC1722639 9797670

[8] Pflugfelder SC, Tseng SC, Sanabria O, Kell H, Garcia CG, Felix C (1998). Evaluation of subjective assessments and objective diagnostic tests for diagnosing tear-film disorders known to cause ocular irritation. Cornea.

[9] Halberg GP, Berens C. Standardized Schirmer tear test kit. Am J Ophthalmol. 1961; 10.1016/0002-9394(61)91827-X. 10.1016/0002-9394(61)91827-x 13710674

[10] Hamor RE, Roberts SM, Severin GA, Chavkin MJ. Evaluation of results for Schirmer tear tests conducted with and without application of a topical anesthetic in clinically normal dogs of 5 breeds. Am J Vet Res. 2000; 10.2460/ajvr.2000.61.1422. 10.2460/ajvr.2000.61.1422 11108191

[11] van der Woerdt A, Adamcak A. Comparison of absorptive capacities of original and modified Schirmer tear test strips in dogs. J Am Vet Med Assoc. 2000; 10.2460/javma.2000.216.1576. 10.2460/javma.2000.216.1576 10825943

[12] Hawkins EC, Murphy CJ (1986). Inconsistencies in the absorptive capacities of Schirmer tear test strips. J Am Vet Med Assoc.

[13] Mishima S, Gasset A, Klyce SD, Baum JL (1966). Determination of tear volume and tear flow. Investig Ophthalmol.

[14] Gelatt KN, MacKay EO, Widenhouse C, Widenhouse TS, Stopek JB. Effect of lacrimal punctal occlusion on tear production and tear fluorescein dilution in normal dogs. Vet Ophthalmol. 2006; 10.1111/j.1463-5224.2005.00430.x. 10.1111/j.1463-5224.2005.00430.x 16409241

[15] Binder DR, Herring IP. Evaluation of nasolacrimal fluorescein transit time in ophthalmically normal dogs and nonbrachycephalic cats. Am J Vet Res. 2010; 10.2460/ajvr.71.5.570. 10.2460/ajvr.71.5.570 20433384

[16] Patton TF, Robinson JR. Influence of topical anesthesia on tear dynamics and ocular drug bioavailability in albino rabbits. J Pharm Sci. 1975; 10.1002/jps.2600640215. 10.1002/jps.2600640215 1127583

[17] Clinch TE. Schirmer’s test. Arch Ophthalmol. 1983; 10.1001/archopht.1983.01040020385009.

[18] Savini G, Prabhawasat P, Kojima T, Grueterich M, Espana E, Goto E. The challenge of dry eye diagnosis. Clin Ophthalmol. 2008; 10.2147/OPTH.S1496. 10.2147/opth.s1496 PMC2698717 19668387

