# Correction to: Photography-based method for assessing fluorescein clearance test in dogs

**DOI:** 10.1186/s12917-018-1632-8

**Published:** 2018-10-08

**Authors:** Arianne Pontes Oriá, Miriam Flores Rebouças, Emanoel Martins Filho, Francisco de Assis Dórea Neto, Ana Cláudia Raposo, Lionel Sebbag

**Affiliations:** 1 School of Veterinary Medicine and Zootechny, Federal University of Bahia, UFBA, Salvador, Bahia Brazil; 2 Department of Veterinary Clinical Sciences, College of Veterinary Medicine, Iowa State University, Ames, Iowa, USA

## Correction

The original article [1] contained an error whereby the respective legends of Figs. 2 and 3 were mistakenly interchanged. This error has now been amended.
